# Tissue response in hemisectioned primary mandibular second molars

**DOI:** 10.2340/aos.v85.46272

**Published:** 2026-07-01

**Authors:** Jenny Öhman, Vini Rughwani, Julia Naoumova

**Affiliations:** a Department of Oral Medicine and Pathology, Institute of Odontology, Sahlgrenska Academy, University of Gothenburg, Gothenburg, Sweden; b Department of Clinical Pathology, Sahlgrenska University Hospital, Gothenburg, Sweden; c Institute of Odontology, Sahlgrenska Academy, University of Gothenburg, Gothenburg, Sweden; d Specialist Clinic of Orthodontics, University Dental Care, Public Dental Service, Region Västra Götaland, Gothenburg, Sweden; e Department of Orthodontics, Institute of Odontology, Sahlgrenska Academy, University of Gothenburg, Gothenburg, Sweden

**Keywords:** Congenitally missing premolars, hemisection, prolonged pulp exposure, reparative dentinogenesis, histopathological features

## Abstract

**Objective:**

Congenital absence of mandibular second premolars occur in 2.91–3.22% of individuals, with nearly half of cases being bilateral. Interceptive hemisection of primary second molars promotes mesial drift but exposes the pulp for an extended period. This study investigates histopathological responses of pulp and associated mineralized tissues.

**Material and methods:**

Children aged 7–12 in Skaraborg region, Sweden, were screened for over-retained primary mandibular second molars as part of a single-center, blinded, prospective split-mouth randomized controlled clinical trial comparing hemisection with extraction. Mesial roots of 21 hemisected teeth were collected for histopathological analysis of inflammatory infiltrate, pulpal necrosis, fibrosis, and hard tissue formation.

**Results:**

Mean interval between distal and mesial root extraction was 11.54 ± 4.3 months. Histopathological analysis revealed no pulpal inflammation in 14% of specimens, acute/subacute inflammation in 38%, chronic inflammation in 48%, and necrosis in 24%. Reparative tertiary dentin with capping occurred in 48% and without capping in 33%. Necrotic mineralized tissue appeared in one specimen. Three patients reported symptoms during follow-up.

**Conclusion:**

Prolonged pulp exposure following hemisection induced tertiary dentinogenesis, maintaining pulp vitality for up to 17 months. Despite histological evidence of inflammation or necrosis, most cases remained asymptomatic, indicating remarkable regenerative capacity of primary pulp–dentin complex.

## Introduction

A meta-analysis conducted in 2004 on Caucasian populations in North America, Australia, and Europe reported that the prevalence of agenesis of mandibular second premolars ranged from 2.91 to 3.22%, and that the condition was bilateral in 43.5–47.7% of affected individuals [1].

Prolonged retention of primary second molars without permanent successors frequently presents with delayed root resorption and infraocclusion. This submergence has the potential to compromise arch integrity by causing adjacent tooth tipping and super-eruption of the tooth in the opposing arch [2–5]. A range of management strategies are available for such cases. One treatment option is to perform hemisection of the retained primary molar to facilitate the mesial migration of the first permanent molar during the eruption of the second permanent molar [3, 6].

Tooth hemisection is a procedure that entails the separation and extraction of a single root and its associated crown segment from a multi-rooted tooth [7]. In the context of permanent dentition, it is indicated when decay or a vertical root fracture is confined to a single root [8]. In primary or mixed dentition, hemisection is employed in cases of congenitally missing second premolars with over-retained primary second molars [9]. Unlike full extraction, which commonly results in vertical and buccolingual alveolar bone loss, hemisection during orthodontic treatment is thought to preserve bone integrity. The retained distal segment functions as a supplementary anchorage for the first permanent molar, thereby facilitating orthodontic management [8]. The technique involves buccolingual sectioning of the mandibular primary second molar and the removal of the distal half, which exposes the pulp to the oral cavity and facilitates spontaneous mesial drift of the first permanent molar. If mesial migration is hindered by the retained mesial portion, this is subsequently removed to allow continued movement and space closure [9].

A tooth is a complex organ consisting of dental pulp, a specialized connective tissue enclosed within the central cavity of a tooth, surrounded by dentin. The pulp plays a crucial role in providing nourishment, sensory function, and regenerative potential [10]. The pulp chambers of primary teeth are proportionally larger than those of permanent teeth. Furthermore, the pulp horns in primary teeth extend closer to the dentin–enamel junction (DEJ), which increases the risk of accidental pulp exposure during operative procedures [11].

A study by Fox et al. [12] identified several similarities and differences in the histological structures of the pulp of primary and permanent teeth. The odontoblast arrangement in primary teeth is comparable to that in permanent teeth, with columnar odontoblasts found in the coronal region, cuboidal odontoblasts in the middle region, and flattened odontoblasts in the apical pulp. Cuboidal and flattened odontoblasts demonstrate reduced metabolic activity, indicating the functional necessity of coronal odontoblasts being more responsive to external stimuli. Apical odontoblasts, on the other hand, are less involved in dentinogenesis [12, 13]. Additionally, the zone of Weil, otherwise known as the cell-free zone is less pronounced in primary teeth. The cell-rich zone in primary teeth contains a higher number of undifferentiated mesenchymal cells and fibroblasts, contributing to a greater reparative capacity [12, 14].

Traumatic injuries are among the leading causes of pulp exposure in both primary and permanent teeth, second only to caries invasion. The histological response of dental pulp to trauma differs significantly between primary and permanent teeth. Primary teeth typically exhibit a significant inflammatory response following injury, attributable to their increased vascularity within the mid-coronal region [15]. This heightened response is facilitated by an abundance of undifferentiated mesenchymal cells, which enhance the formation of reparative dentin and promote healing [13, 14, 16]. Although permanent teeth possess the capacity to undergo reparative dentin formation, this process occurs at a comparatively reduced rate. The structured organization of odontoblasts and other cellular components contributes to a more regulated healing process [13, 14, 17].

Mild injury to a tooth has been shown to preserve the viability of primary odontoblasts, which deposits reactive tertiary dentin with tubular continuity to the primary and secondary dentin [18]. In contrast, more severe injury results in the loss of odontoblast viability and the release of bioactive dentin extracellular matrix components. This process initiates recruitment and differentiation of pulp-resident stem/progenitor cells into odontoblast-like cells, leading to reparative tertiary dentin deposition and dentinal bridge formation at sites of pulpal exposure [19, 20].

To the best of our knowledge, no previous study has evaluated the histopathological response of the pulp and mineralized tissue in hemisectioned primary mandibular second premolars with prolonged exposure of the mesial portion of the tooth to the oral environment.

This study aims to explore and describe the histopathological changes and tissue response of the exposed pulp and mineralized tissue in hemisectioned primary teeth.

## Materials and methods

This study is part of a larger single-center, blinded, prospective, split-mouth randomized controlled clinical trial which was conducted to evaluate the effectiveness of hemisection compared to conventional extraction in cases of over-retained primary second molars [21]. The study was conducted in accordance with the principles outlined in the Declaration of Helsinki and received ethical approval (Dnr: 558-17). Written informed consent and assent were obtained from participants and their parents or guardians.

Children aged 7–12 years were screened for over-retained second primary molars at nine public dental clinics in the Skaraborg region between 2017 and 2020. Those meeting the inclusion criteria were referred to the Orthodontic clinic in Skövde/Falköping, Sweden. The absence of these premolars was detected during routine dental examinations using intraoral radiographs. The inclusion criteria for admitting participants to the study were bilateral agenesis of permanent mandibular second premolars, bilateral persisting deciduous second mandibular molars, and bilateral presence of unerupted mandibular second permanent molars. The exclusion criteria were Angle Class II:2 malocclusion (as mandibular extractions could potentially deepen the bite), generalized spacing in the mandibular arch (defined as ≥ 8 mm of spacing measured from the mesial right primary second molar to the mesial left primary second molar), or cleft lip and palate or other craniofacial syndromes.

### Hemisection

**Figure 1:**
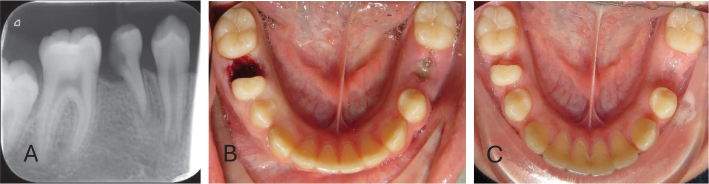
Long-term follow up after hemisection of a primary second molar. (A) Radiograph showing the hemisection and extraction of the distal root of the right primary second molar. (B) A clinical view of the hemisectioned tooth. (C) Clinical status at 64 weeks post-hemisectioning, showing no clinical signs of inflammation.

Local anesthesia (xylocaine-adrenaline injection (20 mg/mL + 12.5 μg/mL Dentsply Pharmaceuticals) was administered prior to the procedure. The deciduous mandibular second molar was sectioned through the furcation using a Zekrya surgical bur. Subsequently, the distal root and crown were extracted using extraction forceps (Figure 1A). The tooth’s mesial half remained in the oral cavity with exposed pulp, without any surgical dressing or endodontic intervention. Hemostasis was achieved using a surgical compress with applied pressure (Figure 1B). Postoperative instructions were provided to patients, including recommendations for analgesic use if necessary.

Participants were recalled every 8 weeks to measure the space between the distal surface of the first permanent molar and the mesial surface of the retained primary molar. Once this space measured less than 2 mm (Figure 1C), the mesial portion of the deciduous molar was extracted using the same procedure. Patients reporting pain after hemisection received Intermediate Restorative Material (IRM) restorations on the mesial half of the tooth. The time interval between extraction of the distal and mesial halves was calculated using an online omni calculator (https://www.omnicalculator.com/everyday-life/months-between-dates), and the meantime interval was recorded.

### Histopathological preparation and evaluation

Immediately after extraction, the teeth were preserved and fixed in 4% buffered formaldehyde (Histolab, Gothenburg, Sweden) for a minimum of 48 hours. The samples were then rinsed in running tap water for 1 hour before decalcification in 10% formic acid (Histolab, Gothenburg, Sweden) with stirring for 4 days. Following decalcification, the tooth was sectioned mesio-distally through the pulp.

The teeth were then dehydrated overnight, paraffin-embedded and sectioned in 4-micrometer-thick sections at two levels and thereafter auto-stained with Mayers hematoxylin and eosin (Histolab, Gothenburg, Sweden). Whole-slide imaging at x20 magnification (Hamamatsu NanoZoomer s210) was performed and used to assess the histopathological changes such as the severity of the inflammatory infiltrate, pulpal necrosis, fibrosis and hard tissue formation. The histopathological assessments were conducted in a blinded manner by a senior oral pathologist and were repeated after an interval of 6 weeks. In cases of discrepancy between the first and second assessments, consensus was reached through discussion among the co-authors (V.R, J.Ö). Variables related to vital and necrotic soft tissue and the type of inflammation were mutually exclusive; however, all other variables could be present in the same tooth.

Definitions of histopathological variables:

- Vital soft tissue was defined as an intact odontoblastic layer without necrosis.
- Necrotic soft tissue was defined as absence of an intact odontoblastic layer with histological signs of necrosis.
- Chronic inflammation was defined as an infiltrate composed of lymphocytes and/or plasma cells.
- Acute or subacute inflammation was defined as an infiltrate containing lymphocytes, plasma cells, and/or neutrophils.
- Fibrosis was defined as collagenous hyperplasia of connective tissue and recorded dichotomously (yes/no).
- Reparative dentin formation was recorded dichotomously (yes/no).
  - Dentin bridging in pulp-capped teeth was defined as reparative dentin deposition resulting in complete enclosure of the pulpal cavity.
  - Dentin bridging in non-pulp-capped teeth was defined as reparative dentin deposition resulting in partial enclosure of the pulpal cavity.

## Results

A total of 21 patients (10 males and 11 females) were included in the study, yielding 21 caries-free hemisected teeth for histopathological evaluation. The overall mean age of the participants was 9.86 ± 1.39 years, with mean ages of 9.36 ± 1.70 years for males and 9.45 ± 0.93 years for females.

The mean interval between the extraction of the distal and mesial halves of the tooth was 11.54 ± 4.3 months. Of the 21 patients, 18 did not report any pain or sensitivity after hemisection. Of the remaining three patients, one experienced pain during mastication 2 weeks after hemisection but did not require any intervention and remained symptom-free thereafter. Another patient reported pain during mastication 14 weeks post-hemisection. An IRM restoration (IRM Caps, Dentsply) was placed, to alleviate the discomfort, and avoid emergency extraction of the mesial half. The third patient returned 1 week after hemisection, reporting symptoms of pain and sensitivity during mastication and tooth brushing. An IRM restoration was provided at that visit. However, 9 weeks later, the patient presented again with similar symptoms, necessitating the extraction of the mesial half of the tooth.

**Figure 2:**
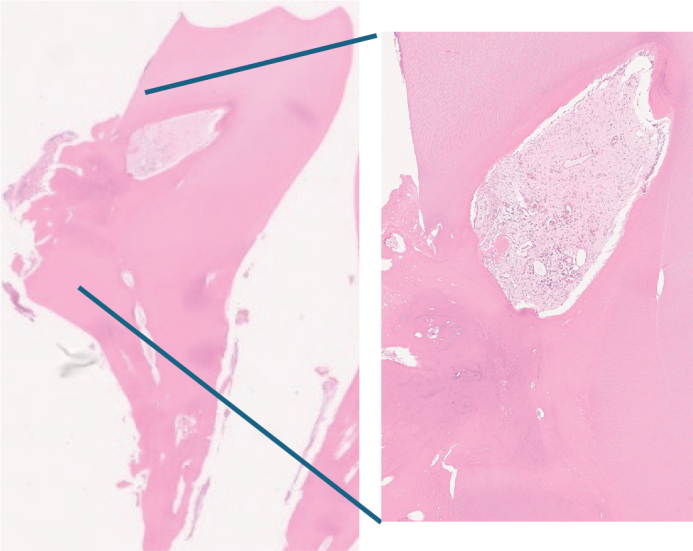
Histopathological section shows the formation of a reparative dentin bridge over a vital pulp with mild fibrosis and minimal inflammation in the mesial half of a hemisected primary mandibular second molar.

**Figure 3:**
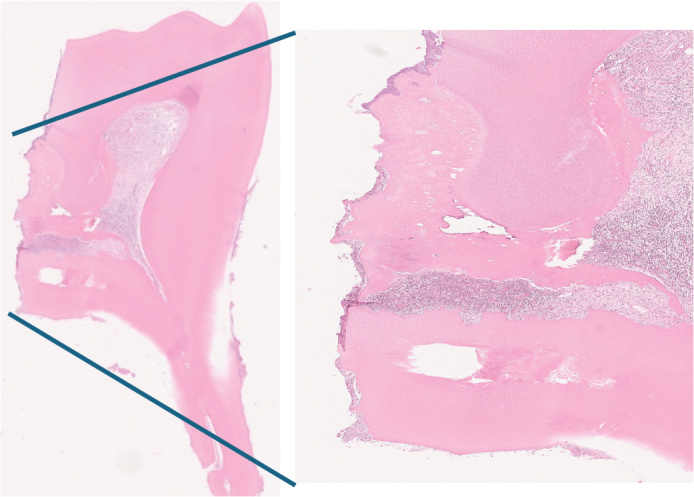
Histopathological section illustrating partial dentin bridging that covers fibrotic pulp tissue with severe subacute inflammation in the mesial half of a hemisected primary mandibular second molar.

**Figure 4:**
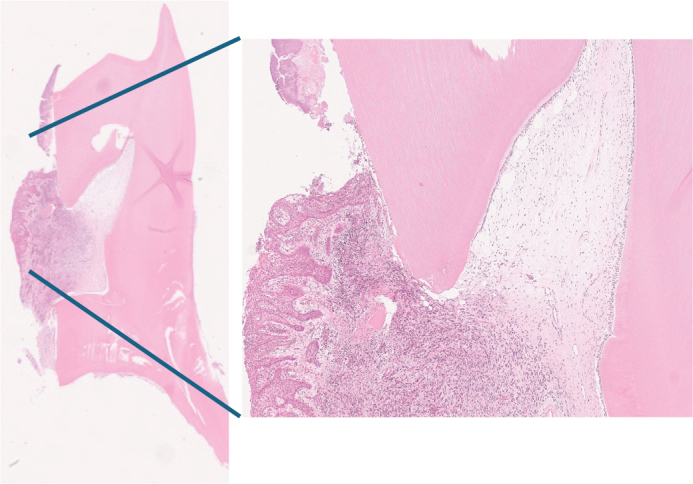
Histopathological section illustrating mucosal coverage with moderate chronic inflammation and mild fibrosis of the coronal pulp in the mesial half of a hemisected primary mandibular second molar. Vital pulp tissue and preserved odontoblast lining are visible in the pulp horn.

**Figure 5:**
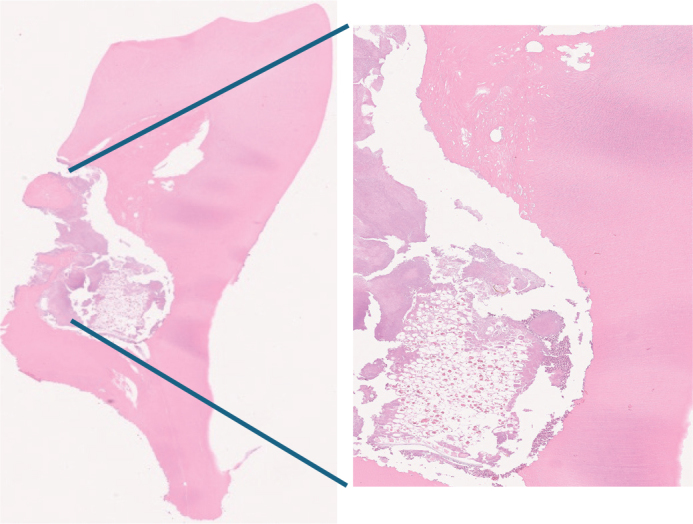
Histopathological section illustrating necrotic pulp with resorption of nonvital atubular reparative dentin as well as secondary and primary dentin in the mesial half of a hemisected primary mandibular second molar.

**Figure 6:**
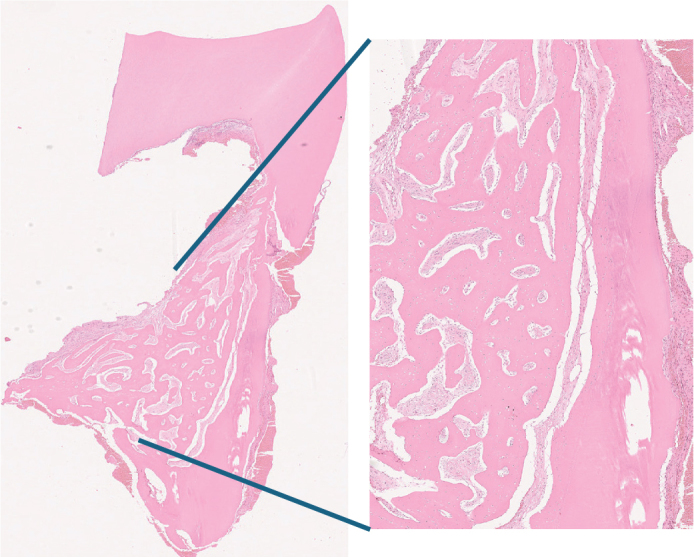
Pulp and primary dentin partially replaced by cementum and immature trabecular bone in the mesial half of a hemisected primary mandibular second molar.

**Figure 7:**
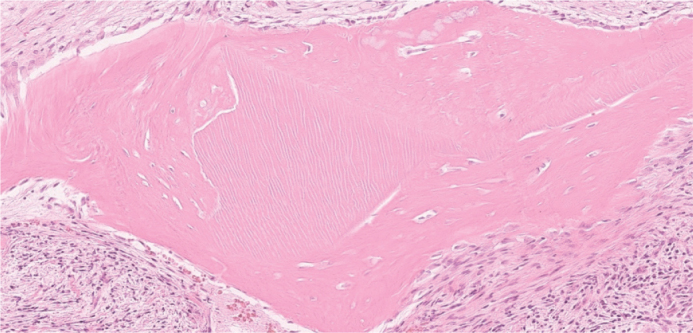
Reparative atubular dentin surrounds resorbed tubular dentin in the mesial half of a hemisected primary mandibular second molar.

Of the hemisected teeth, 14% showed no pulpal inflammation, 38% presented with acute or subacute pulpal inflammation, 48% showed chronic pulpal inflammation, 24% necrosis of the pulp and 66.7% remained with vital pulp. In the majority of teeth (81%), atubular mineralized tissue without an odontoblast lining was observed, possibly representing tertiary reparative dentin formation (Table 1). Dentin bridging with overcapping of the pulp (Table 1 and Figure 2) was observed in 48% of specimens, while reparative tertiary dentin formation without overcapping (Table 1 and Figure 3) was observed in 33% of specimens. A mucosal covering lining the exposed pulpal tissue was noted in 10 cases (48%) (Table 1 and Figure 4). Necrotic reparative tertiary dentin and necrotic secondary dentin were observed only in one specimen (Table 1 and Figure 5). In one case, the dentin and pulpal tissue were almost completely replaced by cementum and newly formed trabecular bone (Figure 6). Occasionally, reparative atubular tertiary dentin was observed surrounding areas of tubular secondary dentin that had undergone partial resorption (Figure 7).

**Table 1:** Pulpal and hard tissue changes observed in mesial halves of the hemisected teeth.

**Soft tissue**	
**Histopathological observation**	***N* (%)**
No inflammation	3/21 (14)
Acute or subacute inflammation	8/21 (38)
Chronic inflammation	10/21 (48)
Necrosis	5/21 (24)
Fibrosis	18/21 (86)
Vital pulp (with or without inflammation)	14/21 (66.7)
**Hard tissue**	
**Histopathological observation**	***N* (%)**
Reparative dentin formation	17/21 (81)
• Dentin bridging with pulp capping	10/21 (48)
• Dentin bridging without pulp capping	7/21 (33)
Necrotic reparative dentin	1/21 (5)

In the two mesial roots treated with IRM, vital pulp tissue without signs of necrosis was observed in both specimens together with mild fibrosis. One specimen demonstrated subacute inflammation and partial tertiary dentin bridging, whereas the other exhibited mild chronic inflammation without tertiary dentin formation. No distinct histological differences were identified when compared with the remaining samples, nor were any clear common features between the two specimens.

Observations of the exposed mesial segments revealed a broad range of pulpal responses. The occurrence of necrosis was documented as early as 6.9 months and as late as 20.4 months post-hemisection. Conversely, the maximum duration for which a segment remained vital – exhibiting only minor inflammatory changes – was 17.1 months.

## Discussion

The present study offers novel insights into the histopathological responses of pulpal and hard tissues following the hemisection of primary mandibular second molars with prolonged pulp exposure to the oral cavity. The primary objective of the histological analysis was to evaluate tissue reactions – both soft and hard – to prolonged pulp exposure rather than to evaluate clinical symptoms. This investigation is a part of a larger clinical trial, for which 21 samples were collected, processed, stained and examined microscopically. Midway through recruitment for the clinical trial, histological analysis was carried out to explore the underlying reasons for the absence of pain following hemisection in a large number of participants. In a related clinical trial comparing postoperative pain following conventional extraction and hemisection, Abdul Jabbar et al. reported that nine out of 41 participants (22%) experienced pain in the mesial root following hemisection which was significantly higher than conventional extraction [21]. In the present subsample, 20 out of 21 participants exhibited pulp exposure for up to 1 year without developing significant clinical complications such as sensitivity or pain that required emergency extraction of the mesial root. Physiologically, the vascular network of the dental pulp is most pronounced in the coronal region. However, because the pulp is encased within a rigid, non-compliant structure, bacterial invasion from carious exposure typically induces inflammation, pain, circulatory stasis, subsequently leading to necrosis. In contrast, direct exposure to the oral cavity in hemisected teeth may permit drainage of inflammatory exudates, enabling better nutrient exchange and supporting the maintenance of essential physiological functions and wound healing [22].

Of the 21 selected patients, three reported experiencing pain from the mesial root. However, only one patient reported experiencing pain or sensitivity severe enough to necessitate extraction of the mesial half of the tooth. This finding is consistent with the observations by Valencia et al. (2009), who noted that the partial removal of pulp tissue alters both pulpal sensitivity and the inflammatory response. Anatomically, the apical foramen is traversed by nerve bundles composed predominantly of unmyelinated fibers, with a smaller proportion of myelinated fibers. The stimulation of these unmyelinated fibers has been demonstrated to reduce pulpal blood flow, which may contribute to a decreased nociceptive response [23].

Histologically, nearly half of the specimens in this study (48%) exhibited chronic inflammation, while 38% demonstrated acute or subacute inflammatory responses. This inflammatory response triggered the development of granulation tissue, characterized by neovascularization and a notable absence of nerve endings at the surface. Remarkably, the pulp maintained its vitality even after almost a year of exposure to the oral environment. These findings contrast with those reported by Valencia et al. [24], who observed no indication of inflammation or necrosis following 4 months of hemisection. However, Valencia et al. (2009) subsequently observed chronic pulpal inflammation 8 months post-exposure, which was associated with granulation tissue formation. This is consistent with our findings, in which prolonged exposure of the pulpal tissue was associated with an increased risk of irreversible tissue damage, including pulpal fibrosis and necrosis.

In the present study, pronounced production of tertiary reparative dentin was observed, forming dentinal bridges that covered the pulp tissue in the majority of hemisectioned teeth. Similar findings have previously been reported in permanent teeth treated with materials such as MTA (mineral trioxide aggregate) following pulp exposure [25–29]. These observations suggest that pulpal responses to MTA capping involve the proliferation and migration of progenitor cells, followed by their differentiation into odontoblast-like cells – a mechanism that is essentially comparable to that induced by calcium hydroxide. The greater reparative capacity of primary teeth is likely due to their higher proportion of undifferentiated mesenchymal cells and fibroblasts, enabling such responses to occur. The findings of this study demonstrate the histopathological reactive changes due to the intrinsic regenerative capacity of primary teeth even in the absence of bioactive materials. The mechanisms of tertiary dentinogenesis have received considerable interest in the context of repairing and regenerating the dentin–pulp complex in many previous studies [30–32]. However, the mechanisms by which these cells differentiate and secrete reparative dentin bridges in the absence of a basement membrane or epithelium still remain to be fully elucidated.

No hypercementosis was observed in the apical portions of the root in any of the patients in this study. This coincides with the findings of Valencia et al. (2009) who also reported no hypercementosis in response to hemisection of primary mandibular second molars in participants aged 8–11 years. However, Saad et al. (1997) observed thickening of the cementum on the root surface of primary mandibular second molars in participants aged 14–40. In the present study, a continuity was noted between the cementum from the coronal part of the root and the formation of the dentin bridge. Histologically, it is difficult to differentiate between atubular reparative dentin and reactive newly formed reactive cementum in the interradicular area. Cementum in primary molars is typically thin; however, in response to traumatic injury, activation of cementoblasts seems to occur, and the development of hypercementosis may represent a reparative mechanism aimed at managing localized inflammation, particularly in the interradicular area. This supports the maintenance of pulpal vitality. Additionally, in the context of a hemisectioned primary molar, hypercementosis of the retained mesial root may be a physiological response to increased functional loading, contributing to reinforcing the root structure and stability [33]. Interestingly, in one of our cases, we observed newly formed immature trabecular bone and cementum formation, replacing pulpal and dentin tissue. A similar phenomenon has been described in a previous case report of a traumatically intruded primary incisor [34]. Further studies are required to better understand the biological mechanisms involved and how constituents may influence cellular behavior in the dentin–pulp complex in primary teeth.

### Limitations

This study represents an analysis of a small subsample from a larger clinical trial in which a higher proportion of participants reported pain following hemisection. Although evaluating all available samples histopathologically would have provided a more comprehensive understanding of the findings, substantial deviations from the current observations are not anticipated. Furthermore, the present analysis relied exclusively on conventional staining methods, which limits the detailed cellular and molecular characterization of the reparative and healing processes. Further research should incorporate larger, multicenter cohorts and employ advanced histological and molecular techniques to elucidate the underlying mechanisms governing reparative dentinogenesis and pulpal healing in primary teeth.

## Conclusion

This study provides insight into the remarkable capacity of pulp–dentin complex to self-repair after exposure to abrupt mechanical trauma, evidenced by substantial reparative tertiary dentin formation following prolonged exposure to the oral cavity. The pulp was observed to maintain its vitality despite being exposed to the oral environment for more than a year. Even in the presence of inflammation or necrosis of soft and hard tissues, no significant clinical complications were observed.

## Geolocation information

The study was conducted in the Region of Västra Götaland, Sweden.

## Data Availability

All data generated or analyzed during this study are included in this article. Further inquiries can be directed to the corresponding author.

## Clinical trial number

This study is a part of a larger single-center, blinded, prospective, split-mouth randomized controlled clinical trial which was registered with https://www.researchweb.org/is/sverige, registration number 967125.

## References

[1] Polder BJ, Van’t Hof MA, Van der Linden FP, Kuijpers-Jagtman AM. A meta-analysis of the prevalence of dental agenesis of permanent teeth. Community Dent Oral Epidemiol. 2004;32(3):217–26. 10.1111/j.1600-0528.2004.00158.x 15151692

[2] Sletten DW, Smith BM, Southard KA, Casko JS, Southard TE. Retained deciduous mandibular molars in adults: a radiographic study of long-term changes. Am J Orthod Dentofacial Orthop. 2003;124(6):625–30. 10.1016/j.ajodo.2003.07.002 14666074

[3] Valencia R, Saadia M, Grinberg G. Controlled slicing in the management of congenitally missing second premolars. Am J Orthod Dentofacial Orthop. 2004;125(5):537–43. 10.1016/j.ajodo.2003.05.009 15127021

[4] Kurol J, Thilander B. Infraocclusion of primary molars with aplasia of the permanent successor. A longitudinal study. Angle Orthod. 1984;54(4):283–94. 10.1093/ejo/6.4.277 6594959

[5] Iraqi G, Helal N, Arafa A, Helal F. Retained primary molars and related reasons in Umm Al-Qura University, Makkah: a retrospective study. Open Dent J. 2019;13(1):190–5. 10.2174/1874210601913010190

[6] Williams R, Park JH, Chae JM, Vaden JL. The congenitally missing second premolar: space closure. A viable option. Am J Orthod Dentofacial Orthop. 2020;157(4):571–83.e16. 10.1016/j.ajodo.2019.10.015 32241364

[7] Alqahtani SM. Tooth hemisection. Case study and literature review. Int J Med Dent. 2019;23(2):272–6.

[8] Northway WM. The nuts and bolts of hemisection treatment: managing congenitally missing mandibular second premolars. Am J Orthod Dentofacial Orthop. 2005;127(5):606–10. 10.1016/j.ajodo.2004.12.001 15877042

[9] Radke U, Kubde R, Paldiwal A. Hemisection: a window of hope for freezing tooth. Case Rep Dent. 2012;2012:390874. 10.1155/2012/390874 22928121 PMC3426179

[10] Miller N. Ten Cate’s oral histology. 8th ed. London: Nature Publishing Group UK; 2012. p. 157–92.

[11] Costa VPP, de Queiroz IQD, Lia ÉN. Primary and Permanent Dentitions: Characteristics and Differences. In: Coelho Leal S, Takeshita E, editors. Pediatric Restorative Dentistry. Cham: Springer; 2019. 10.1007/978-3-319-93426-6_3

[12] Fox AG, Heeley JD. Histological study of pulps of human primary teeth. Arch Oral Biol. 1980;25(2):103–10. 10.1016/0003-9969(80)90084-9 6931557

[13] Nylen M. Electron microscopic studies of odontogenesis. J Indiana Dent Ass. 1960;39:406–21.

[14] Kubota K, Kubota J. On the formation of the so-called cell-rich zone in the human dental pulp. Okajimas Folia Anat Jap. 1961;37(1):29–47. 10.2535/ofaj1936.37.1_29

[15] Rodd HD, Boissonade FM. Vascular status in human primary and permanent teeth in health and disease. Eur J Oral Sci. 2005;113(2): 128–34. 10.1111/j.1600-0722.2005.00193.x 15819818

[16] Raslan N, Wetzel WE. Exposed human pulp caused by trauma and/or caries in primary dentition: a histological evaluation. Dent Traumatol. 2006;22(3):145–53. 10.1111/j.1600-9657.2006.00410.x 16643290

[17] Andreasen FM, Kahler B. Pulpal response after acute dental injury in the permanent dentition: clinical implications – a review. J Endod. 2015;41(3):299–308. 10.1016/j.joen.2014.11.015 25601716

[18] Murray PE, About I, Lumley PJ, Smith G, Franquin JC, Smith AJ. Postoperative pulpal and repair responses. J Am Dent Assoc. 2000; 131(3):321–9. 10.14219/jada.archive.2000.0175 10715923

[19] Smith AJ, Scheven BA, Takahashi Y, Ferracane JL, Shelton RM, Cooper PR. Dentine as a bioactive extracellular matrix. Arch Oral Biol. 2012;57(2):109–21. 10.1016/j.archoralbio.2011.07.008 21855856

[20] Smith AJ, Patel M, Graham L, Sloan AJ, Cooper PR. Dentine regeneration: key roles for stem cells and molecular signalling. Oral Biosci Med. 2005;2(2/3):127–32.

[21] Abdul Jabbar S, Nawaia S, Rughwani V, Hansen K, Naoumova J. Hemisection versus conventional extraction as interceptive treatment in congenitally missing mandibular second premolars: a randomised controlled split-mouth trial. Eur J Orthod. 2025;47(4):cjaf043. 10.1093/ejo/cjaf043 40501277 PMC12159412

[22] Haskell EW, Stanley HR. Vital hemisection of a mandibular second molar: a case report. J Am Dent Assoc. 1981;102(4):503–6. 10.14219/jada.archive.1981.0131 6938582

[23] Valencia R, Espinosa R, Torres MA, Saadia M. Long-term histological response of hemisectioned exposed primary pulps: an in vivo study. J Clin Pediatr Dent. 2009 Fall;34(1):19–24. 10.17796/jcpd.34.1.n51v8102r8j7821m 19953804

[24] Edwall L, Kindlová M. The effect of sympathetic nerve stimulation on the rate of disappearance of tracers from various oral tissues. Acta Odontol Scand. 1971;29(4):387–400. 10.3109/00016357109026527 5289328

[25] Kuratate M, Yoshiba K, Shigetani Y, Yoshiba N, Ohshima H, Okiji T. Immunohistochemical analysis of nestin, osteopontin, and proliferating cells in the reparative process of exposed dental pulp capped with mineral trioxide aggregate. J Endod. 2008;34(8):970–4. 10.1016/j.joen.2008.03.021 18634929

[26] Fernandes AM, Silva GA, Lopes N, Jr., Napimoga MH, Benatti BB, Alves JB. Direct capping of human pulps with a dentin bonding system and calcium hydroxide: an immunohistochemical analysis. Oral Surg Oral Med Oral Pathol Oral Radiol Endod. 2008;105(3):385–90. 10.1016/j.tripleo.2007.08.031 18280971

[27] Masuda-Murakami Y, Kobayashi M, Wang X, Yamada Y, Kimura Y, Hossain M, et al. Effects of mineral trioxide aggregate on the differentiation of rat dental pulp cells. Acta Histochem. 2010;112(5):452–8. 10.1016/j.acthis.2009.05.001 19560800

[28] Simon S, Cooper P, Smith A, Picard B, Naulin Ifi C, Berdal A. Evaluation of a new laboratory model for pulp healing: preliminary study. Int Endod J. 2008;41(9):781–90. 10.1111/j.1365-2591.2008.01433.x 18798922

[29] Moghaddame-Jafari S, Mantellini MG, Botero TM, McDonald NJ, Nör JE. Effect of ProRoot MTA on pulp cell apoptosis and proliferation in vitro. J Endod. 2005;31(5):387–91. 10.1097/01.don.0000145423.89539.d7 15851935

[30] Nowicka A, Wilk G, Lipski M, Kołecki J, Buczkowska-Radlińska J. Tomographic evaluation of reparative dentin formation after direct pulp capping with Ca(OH)2, MTA, biodentine, and dentin bonding system in human teeth. J Endod. 2015;41(8):1234–40. 10.1016/j.joen.2015.03.017 26031301

[31] Edanami N, Yoshiba K, Ibn Belal RS, Yoshiba N, Takenaka S, Ohkura N, et al. Role of dystrophic calcification in reparative dentinogenesis after rat molar pulpotomy. Int J Mol Sci. 2025;26(15):7130. 10.3390/ijms26157130 40806263 PMC12346537

[32] Hosoya A, Nakamura H. Ability of stem and progenitor cells in the dental pulp to form hard tissue. Jap Dent Sci Rev. 2015;51(3):75–83. 10.1016/j.jdsr.2015.03.002

[33] Nanci A. Ten Cate's Oral Histology: Development, Structure, and Function. 7th ed. St. Louis (MO): Mosby Elsevier; 2007. p. 411.

[34] Nelson‐Filho P, Borsatto MC, De Oliveira PT, Da Silva RAB. Partial replacement of the dentin–pulp complex by periodontal supporting tissues in a traumatically intruded primary maxillary incisor. Dent Traumatol. 2008;24(5):553–5. 10.1111/j.1600-9657.2007.00549.x 18821962

